# Experience with modified radical mastectomy in a low-income country: a multi-center prospective observational study

**DOI:** 10.1186/s12893-021-01374-1

**Published:** 2021-10-20

**Authors:** Giziew Bawoke, Segni Kejela, Abebe Alemayehu, Girmaye Tamirat Bogale

**Affiliations:** Department of Surgery, College of Health Sciences, Addis Ababa University, Addis Ababa, Ethiopia

**Keywords:** Modified radical mastectomy, Early postoperative complications, Neoadjuvant chemotherapy, Breast cancer

## Abstract

**Background:**

Modified radical mastectomy is the procedure of choice in centers with little to no radiotherapy services. Studying the in-hospital outcome and complications associated with the procedure is important in low-income countries.

**Methods:**

This is a multi-center prospective observational study involving all patients operated with modified radical mastectomy with curative intent.

**Results:**

A total of 87 patients were studied with 10.3% of which were male and 54% were between the age of 30–49 years. Clinical stage IIB and IIIA were reported in 33 (37.9%) and 25 (28.7%) respectively and 62.1% had clinically positive lymph nodes at presentation. All of the studied patients underwent curative surgery, with an average lymph node dissection of 10.2 ± 0.83. Seroma rate was 17.2% and was significantly associated with diabetes (AOR: 6.2 (CI 1.5–8.7)) and neoadjuvant chemotherapy (AOR: 8.9 (CI 1.2–14.2)). Surgical site infection occurred in 14.9% and was significantly associated with Retroviral infections (AOR: 4.2 (CI 2.1–5.8)) and neoadjuvant chemotherapy (AOR: 1.8 (CI 1.3–3.9)). No in-hospital mortality occurred during the course of the study.

**Conclusion:**

Seroma rate was lower than published studies while surgical site infections rate was higher. Neoadjuvant chemotherapy was associated with increase in seroma and surgical site infection rates. Additionally, diabetes increased the rate of seroma. Surgical site infections were higher in patients with retroviral infections.

## Introduction

The low and middle income countries are responsible for more than 60% of breast cancer related global annual mortality. This burden is even more pronounced in Sub-Saharan Africa [1, 2]. In Ethiopia, as in all Sub-Saharan nations, breast cancer is a concerning public health problem, contributing to more than 30% of cancer diagnosis in women [3].

Prognosis of breast cancer patients in low income countries like Ethiopia is poor owing, in part, to the late clinical stages of the disease at diagnosis [4–6]. Additionally, advanced diagnostic and therapeutic modalities are largely lacking [7]. For instance, radiotherapy services accessibility has fallen short of the international atomic agency recommendations of four machines per 1 million population in all African countries, with most reporting one or no machine [8]. Ethiopia, similarly, has one machine for a population of 118 million, although efforts are underway to decrease this ratio significantly [9, 10]. This has forced most surgeons in these setups to avoid breast-conserving surgeries and instead perform mastectomy to avoid the need for radiation therapy in most patients regardless of the stage at diagnosis [11].

The lack of available radiotherapy facilities and advanced stage of patients at presentation have made mastectomy the preferred surgery in Ethiopia. Hence, it is imperative to study the in-hospital outcomes of mastectomy in a low-income country to assess its safety with regards to short term complications, and efficacy with regards to the status of oncologic resection.

## Methods

### Study setting

This study was done in four tertiary teaching hospitals in the capital city, Addis Ababa, Ethiopia. The hospitals were Tikur Anbessa Specialized Hospital, Zewditu Memorial Hospital, Yekatit 12 Memorial Hospital and Minilik II Memorial Hospital. These hospitals serve populations both from Addis Ababa and regional states with sub-specialty level surgical service.

### Study design and population

This is a multi-institutional prospective observational study of in-hospital outcomes and complications of patients undergoing Modified radical mastectomy. The study population was all patients undergoing Modified radical mastectomy for curative intent operated during the study period (July 1, 2019, to September 30, 2020) at the four hospitals. All patients undergoing palliative mastectomy and radical mastectomy were excluded from this study.

### Study variables

Independent variables were age, sex, residence, smoking history, comorbidities, body mass index, histologic type, clinical-stage, history of neoadjuvant therapy, level of lymph node dissection, number of positive lymph nodes, and history preoperative prophylactic antibiotics.

Dependent variables were rate of seroma, surgical site infections, hematoma, skin flap necrosis, and perioperative mortality rate.

### Data collection and analysis

Data from the patients were collected using a structured format filled during their in-hospital stay prospectively. All complications were daily evaluated and recorded.

Completeness of the data was assured at each step and entered into the SPSS version 23. Initial descriptive analysis was done for socio-demographic and clinical variables in the study with mean, median, range, and frequency. Then inferential analysis was done using Student t-test and Chi-square depending on the type of the variable, and values in univariable regression found to be less than 0.2 were evaluated in multivariable regression.

### Outcomes

Primary outcomes were the rates of surgical complications such as, seroma, surgical site infections, wound dehiscence, skin flap necrosis and hematoma formation. In addition, associations between preoperative comorbidities, with postoperative complications were assessed.

Secondary outcomes were rate of complete pathologic resection, lymph node harvest rate, level of lymph node dissection, drainage tube removal and length of hospital stay.

### Surgical procedure

The “modified radical mastectomy” procedure done according to the standard recommendations in all study centers. In all patients, elliptical incisions surrounding the nipple-areola complex were made. Then skin flap was raised in the superior direction to the level of the clavicle and in the inferior aspect to the level of the infra-mammary fold. The Pectoralis fascia was raised from the underlying pectoralis muscle completely. The axilla was dissected in levels I and II in all patients. Optional level III dissection was done where there was bulky level II lymphadenopathy or any level III lymphadenopathy. The drain was placed within 2 cm of the surgical wound margin and the wound was closed primarily. The number of drains used (mostly 2) and the method of wound closure were left to the discretion of the operating surgeon. All specimens were subjected to pathology evaluation. There was no single case of immediate breast reconstruction.

### Ethical considerations

Ethical approval was gained from the Institutional Review Board of Addis Ababa University, College of Health Sciences, which is the governing body of health researches under Addis Ababa University. The approval letter was disseminated to all affiliated hospitals for the study and each hospital involved in the study provided approval for the data collection. The study was conducted in accordance to Helsinki declarations, Ethiopian National Research Ethics Guidelines, and Institutional regulations on research ethics.

All participants of the study provided signed written informed consent before initiation of the data collection. All data collected from the patients were kept confidential. No individual outside of the authors had any access to the patients' information.

### Funding and conflict of interest

Funding for this study was acquired from Addis Ababa University, College of health sciences. Funder had no contribution to the study design, study conduct or manuscript preparation, and had no say on conclusion and dissemination of results.

## Results

### Sociodemographic factors of the patients

A total of 87 consecutive patients were evaluated during the course of the study of which 78 (89.7%) were females. The most common age groups were 30–39 years and 40–49 years with 24 (27.6%) and 23 (26.4%) respectively, and a mean age of 45.6 ± 12.5 years. 70 (80.5%) of the patients were from the capital, Addis Ababa, while the rest were from regional states. Hypertension and retro-viral infections were the most common comorbidities, with 12 (13.8%) and 10 (11.5%) respectively. 2 (2.3%) of the patients were obese (BMI > 30 kg/m^2^), while 4 (4.6%) were found to have moderate to severe under-nutrition (BMI < 17 kg/m^2^). 17 (19.5%) of the patients had neoadjuvant chemotherapy (Table 1).

**Table 1:** Sociodemographic and surgical factors of the patients with modified radical mastectomy at four teaching hospitals, Addis Ababa, Ethiopia (n = 87)

No	Variables	Category	Number	Percentage (%)
1	Sex	Male	9	10.3
		Female	78	89.7
2	Age (years)	24–29	8	9.2
		30–39	24	27.6
		40–49	23	26.4
		50–59	21	24.1
		60–69	4	4.6
		70–79	7	8
3	Residence	Addis Ababa	70	80.5
		Regional states	17	19.5
4	Comorbidities	Hypertension	12	13.8
		Diabetes mellitus	7	8
		Retroviral infection	10	11.5
		Others	4	4.6
5	Body Mass index(kg/m^2^)	> 30	2	2.3
		24–29.9	6	6.9
		17–23.99	75	81.6
		< 17	4	4.6
6	Preoperative treatment	Neoadjuvant chemotherapy	17	19.5
		Without neoadjuvant chemotherapy	70	80.5
7	Clinical tumor stage	T1	5	5.7
		T2	31	35.6
		T3	37	42.5
		T4	14	16
8	Lymph node status	Negative	33	37.9
		Positive	54	62.1
9	Level of Lymph node dissection	Level I and II	84	96.6
		Level I–III	3	3.4
10	Number of lymph nodes harvested	≥ 10	45	51.7
		< 10	42	48.3

### Cancer characteristics and surgery

**Fig. 1:**
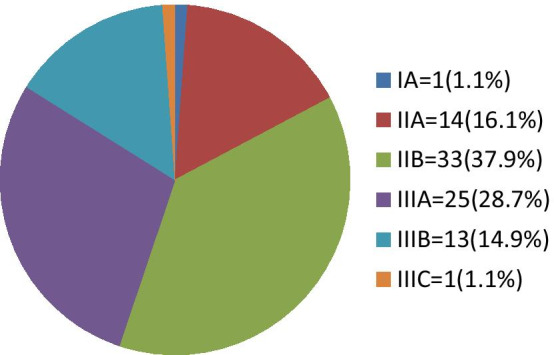
Clinical stage of patients at presentation

With regards to the clinical T stage of the patients, T2 and T3 were the most common tumor stages at 31 (35.6%) and 37 (42.5%) respectively, while T1 and T4 were the least common, contributing to 5 (5.7%) and 14 (16%) respectively, while. At the time of admission for surgery, 54 (62.1%) of the patients had clinically positive lymph nodes (Table 1). The most common stages at diagnosis were AJCC 8 clinical-stage IIB and IIIA at 33 (37.9%) and 25 (28.7%) respectively. Stage IA and IIIC were the least common with 1 patient in each stage. (Fig. 1) Sixteen (94.1%) of the 17 patients residing outside of the capital city had stage III disease, compared to 23 (32.8%) of the 70 patients from Addis Ababa. Compared to 8 (88.9%) of the 9 male patients with stage III disease, 31 (39.7%) of the 78 of all the female patients had stage III disease at diagnosis. All patients had preoperative histologic diagnosis. Ductal carcinoma was reported in 79 (90.8%), and lobular carcinoma in 5 (5.7%). 3 (3.4%) patients had no specific histologic subtype reported.

During surgery, prophylactic preoperative antibiotics were avoided in all patients. All underwent curative surgery with pathologically confirmed clear tumor margins, and with level I and II lymph node dissection in 84 (96.6%), and level I–III in the remaining group of patients. The average number of lymph nodes retrieved were 10.2 ± 0.83 with a range of 2–19. 45 (51.7%) patients had 10 or more lymph nodes harvested. The average number of pathologically positive lymph nodes was 4.71 ± 0.71 with a range of 0–14. At the completion of the surgery, all patients had drainage tubes placed which was removed, on average, on postoperative day 5 with a range of 3–17 days.

### Postoperative complications and regression analysis

**Fig. 2:**
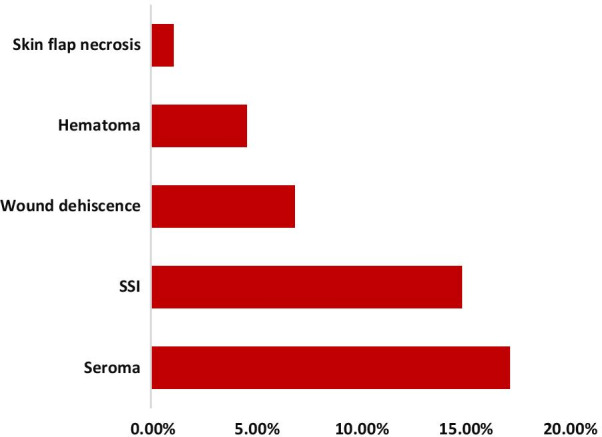
Postoperative complications after modified radical mastectomy

Postoperatively, 25 (28.7%) of the patients had one or more complications. The overall incidence of seroma, the most common postoperative complication, was 15 (17.2%). The rate of surgical site infections was 13 (14.9%). Hematoma and wound dehiscence occurred in 4 (4.6%) and 6 (6.9%) patients respectively. 1 patient had skin flap necrosis (Fig. 2).

In the regression analysis, seroma was positively associated with diabetes (p-value: 0.001), history of neoadjuvant chemotherapy (p-value: 0.006), 10 or more lymph nodes harvested (p-value: 0.006), Body Mass Index of ≥ 24 kg/m^2^ (p-value: 0.001) on the univariable analysis. On multivariable analysis, diabetes (AOR: 6.2 (1.5–8.7), p-value: 0.043) and neoadjuvant chemotherapy (AOR: 8.9 (1.2–14.2), p-value 0.035) were statistically significant in their association with seroma. The number of lymph nodes harvested and BMI had a trend towards statistical significance (AOR: 1.5 (1.11–3.1), p-value: 0.10, and AOR: 1.4 (1.2–2.3), p-value: 0.14 respectively) (Table 2). Univariable analysis showed retroviral infections (RVI) (p-value: 0.001), cigarette smoking (p-value: 0.041) and neoadjuvant chemotherapy (p-value: 0.014), level III lymph node dissection (p-value: 0.041) and presence of seroma (p-value: 0.037) to be positively associated with surgical site infections. On multivariable analysis, RVI (AOR: 4.2 (2.1–5.8), p-value: < 0.001) and history of neoadjuvant chemotherapy (AOR: 1.8 (1.3–3.9), p-value: 0.026) were found to be statistically significant (Table 3). No mortality was reported among the study group during the in-hospital course of the patients.

**Table 2:** Factors associated with seroma

No	Variables	Category	Seroma rate (%)		p-value (univariable analysis)	AOR	p-value
			Seroma	No seroma			
1	Diabetes	Yes	6 (85.7)	1 (14.3)	0.001	6.2 (1.5–8.7)	0.043
		No	9 (11.2)	71 (88.8)			
2	Neoadjuvant	Yes	7 (41.2)	10 (58.8)	0.006	8.9 (1.2–14.2)	0.035
		No	8 (11.4)	62 (88.6)			
3	LN harvest	≥ 10 LN	14 (31.1)	31 (68.9)	0.006	1.5 (1.11–3.1)	0.10
		< 10 LN	1 (2.4)	41 (97.6)			
4	BMI	≥ 24 kg/m^2^	7 (87.5)	1 (12.5)	< 0.001	1.4 (1.2–2.3)	0.14
		< 24 kg/m^2^	8 (10.1)	71 (89.9)			

*LN* lymph nodes, *AOR* adjusted odds ratio, *BMI* Body Mass Index

**Table 3:** Factors associated with surgical site infections

No	Variables	Category	SSI rate (%)		p-value (univariable analysis)	AOR	p-value
			SSI	No SSI			
1	RVI	Yes	8 (80)	2 (20)	< 0.001	4.2 (2.1–5.8)	< 0.001
		No	5 (6.5)	72 (93.5)			
2	Neoadjuvant	Yes	6 (35.3)	11 (64.7)	0.014	1.8 (1.3–3.9)	0.026
		No	7 (10)	63 (90)			
3	LN level	I–III	2 (66.7)	1 (33.3)	0.06	1.16 (1.03–2.5)	0.10
		I and II	11 (13.1)	73 (86.9)			

*RVI* Retroviral infection, *LN* lymph nodes, *SSI* surgical site infections, *AOR* adjusted odds ratio

## Discussion

As far as we know this prospective study is the first of its kind to study early post mastectomy complications in Ethiopia. In this study, just more than 10% of the patients were males and more than 50% were among the age group 30–49 years. Hypertension and RVI were the most commonly reported comorbidities, and two-thirds of the patients had stage IIB and IIIA disease. Level I and II lymph node dissection was performed in more than 96% of the patients. 28.7% of the patients had at least one postoperative complication with seroma and surgical site infections being the most commonly reported complications. Patients with a history of neoadjuvant chemotherapy had 8.9 times higher rate of seroma and 1.8 times higher rate of surgical site infections compared to the up-front surgery group. Diabetes was associated with an increase in seroma rate by more than sixfold. Patients with retroviral infection had 4.2 times higher risk of surgical site infection.

Breast cancer in Ethiopia has consistently been shown to be associated with advanced stage at diagnosis [4, 7, 11]. Concordant reports have been published from other low-income countries [12–14]. Even in a study like ours where only patients with loco-regional disease were included, the proportion of early local stage like stage I is still extremely small. Lack of national screening programs, among other factors, may have contributed to the delay in diagnosis with resultant advanced stage at presentation in low income setups [15]. Published studies have confirmed that advanced stage at diagnosis and lack of appropriate treatment options were the main contributors to the disproportionately high rate of mortality from breast cancer in developing countries [15, 16].

This study found an enigma concerning the association between breast cancer and retroviral infections. In contrast to published bodies of literature reporting lower or equivalent breast cancer rate in patients with retroviral infection compared to general population, our study found that the proportion of breast cancer patients with retroviral infection exceeded 10%, which is far more than the rate of retroviral infections in the capital, 3.4% [17, 18]. We understand that the study was not designed to answer this question. Nonetheless, both population retroviral seropositivity rate and association between breast cancer and retroviral infections need reevaluation.

In our study, no prophylactic antibiotics was used and postoperatively, drainage tube was removed on the 5th postoperative day. Avoidance of prophylactic antibiotics for mastectomy, and drain removal on postoperative day 5 instead of on earlier days, are both supported by the current pieces of evidence [19, 20]. The level of lymph node dissections required during axillary lymph node dissection is set by the NCCN as level I and II anatomical regions of lymph nodes, and 10 lymph node numbers [21]. In our study, we were able to confirm that all patients underwent at least level I and II lymph node dissections and the average number of lymph nodes retrieved were 10.

The rate of seroma for mastectomy patients in other studies has been reported to range between 3 and 85% with an average of 39%, which is higher than the result we have found in our study, which was 17.2% [22, 23]. On the contrary, the rate of surgical site infections, 14.9% in our study, was higher in our patient population compared to published reports which ranged between 2 and 6% [24–26].

Reports indicated older age, Hypertension, high volume of drainage output, serum protein concentration, longer operative time, and lack of patient-controlled analgesia to be risk factors for seroma formation [23, 27–29]. In contrast, in our study, we found a higher seroma rate in patients with a history of diabetes, neoadjuvant chemotherapy, 10 or more lymph node dissection and a higher BMI (> 24 kg/m^2^). Increase in seroma rate in patients treated with neoadjuvant chemotherapy has been reported before [29]. Similarly, the association between a higher BMI and seroma formation has been confirmed in published studies [29, 30]. Likewise, the number of lymph nodes retrieved in mastectomy is reported to be directly associated with rate of seroma formation. This is further affirmed by the report showing lower rate of seroma formation in patients undergoing sentinel lymph node biopsies compared to lymph node dissections [29, 31, 32]. But the association between seroma and diabetes found in our study, is not replicated in any other publication, and requires further study.

We found surgical site infections to be higher in patients treated with neoadjuvant chemotherapy and patients with retroviral infections. The association between RVI and surgical site infections has been reported for patients in orthopedics surgery even though the evidence was not robust [33]. One matched study for major general, orthopedic and gynecologic procedures failed to show an increase in postoperative complications rate except in patients with low CD4 count (50/μL) and high viral load (30,000 c/mL), which showed trends towards statistical significance [34]. In breast cancer surgery, we are not aware of any report on the association between surgical site infection and retroviral infections. Similarly, neoadjuvant chemotherapy has been shown to increase wound complications only in cohort of patients with mastectomy and immediate breast reconstruction [35]. Another review showed an increase in surgical site infections in the adjuvant chemotherapy group compared to neoadjuvant and no systemic therapy group in patients undergoing mastectomy with immediate breast reconstruction [36]. Another study has also disputed any increase in surgical site infections in patients with a history of neoadjuvant chemotherapy in patients with no immediate breast reconstruction [37]. Our finding stands in sharp contrast to available western evidence regarding neoadjuvant chemotherapy. With respect to association between RVI and surgical site infections, Ethiopian seropositive patients are shown to have a higher rate of nutritional deficiencies, higher viral load, and lower CD4 count relative to the western patients [38]. We suspect that the level of immunocompromisation of our patient population might have contributed to the higher rate of surgical site infections. Our study couldn’t establish any association between seroma formation and surgical site infections.

Strengths of this study include, its prospective design and multi-center involvement. The limitations of this study were, lack of further followup for delayed complications like upper extremity lymphedema. Smaller sample size due to a shorter study time of only 14 months can be added as another limitation. Finally, larger sample size and longer study should be conducted to evaluate both short and long term complications of mastectomy. In addition, a study is required to identify the cause for higher rate of surgical site infections.

## Conclusion

Our finding showed that, even though the rate of seroma formation was lower than previous reports, surgical site infections rate was higher than previous studies. History of neoadjuvant chemotherapy was associated with increased seroma and surgical site infection rate. Furthermore, Diabetes, BMI ≥ 24 kg/m^2^ and ≥ 10 lymph node harvested increased the rate of seroma. Surgical site infections were higher in patients with retroviral infections and level III lymph node dissections.

## Data Availability

The data and material for this study would be provided with reasonable request to the corresponding author.

## Abbreviations

AOR: Adjusted odds ratio
BMI: Body Mass Index
CD4: Cluster of differentiation 4
HIV/AIDS: Human immunodeficiency virus/acquired immunodeficiency syndrome
RVI: Retroviral infection
SPSS: Statistical Package for the Social Sciences
